# Suppression of *ABHD2*, identified through a functional genomics screen, causes anoikis resistance, chemoresistance and poor prognosis in ovarian cancer

**DOI:** 10.18632/oncotarget.9951

**Published:** 2016-06-13

**Authors:** Koji Yamanoi, Noriomi Matsumura, Susan K. Murphy, Tsukasa Baba, Kaoru Abiko, Junzo Hamanishi, Ken Yamaguchi, Masafumi Koshiyama, Ikuo Konishi, Masaki Mandai

**Affiliations:** ^1^ Department of Gynecology and Obstetrics, Kyoto University Graduate School of Medicine, Kyoto, Japan; ^2^ Division of Gynecologic Oncology, Department of Obstetrics and Gynecology, Duke University Medical Center, Durham, NC, USA; ^3^ Department of Obstetrics and Gynecology, Faculty of Medicine, KinKi University, Osaka, Japan

**Keywords:** ovarian cancer, anoikis resistance, functional genomics screen, shRNA library

## Abstract

Anoikis resistance is a hallmark of cancer, and relates to malignant phenotypes, including chemoresistance, cancer stem like phenotypes and dissemination. The aim of this study was to identify key factors contributing to anoikis resistance in ovarian cancer using a functional genomics screen. A library of 81 000 shRNAs targeting 15 000 genes was transduced into OVCA420 cells, followed by incubation in soft agar and colony selection. We found shRNAs directed to *ABHD2, ELAC2* and *CYB5R3* caused reproducible anoikis resistance. These three genes are deleted in many serous ovarian cancers according to The Cancer Genome Atlas data. Suppression of *ABHD2* in OVCA420 cells increased phosphorylated p38 and ERK, platinum resistance, and side population cells (p<0.01, respectively). Conversely, overexpression of *ABHD2* decreased resistance to anoikis (p<0.05) and the amount of phosphorylated p38 and ERK in OVCA420 and SKOV3 cells. In clinical serous ovarian cancer specimens, low expression of *ABHD2* was associated with platinum resistance and poor prognosis (p<0.05, respectively). In conclusion, we found three novel genes relevant to anoikis resistance in ovarian cancer using a functional genomics screen. Suppression of *ABHD2* may promote a malignant phenotype and poor prognosis for women with serous ovarian cancer.

## INTRODUCTION

Ovarian cancer is the most lethal cancer among gynecologic malignancies [1]. New treatment strategies are urgently need to help improve prognosis. To this end, it is important to elucidate the detailed molecular characteristics of ovarian cancer. There are several pathological phenotypes of ovarian cancer. High-grade serous ovarian cancer (HGSOC) is the most frequent subtype with a very poor prognosis [2].

Recently, genomic analyses of HGSOC, including The Cancer Genome Atlas (TCGA) project, have begun to shed light on many of the genetic and epigenetic features of these tumors [3]. From TCGA findings, HGSOC is characterized by ubiquitous *TP53* mutations and extensive copy number alterations. However, it is unclear which of the numerous genome-wide genetic changes are involved in the HGSOC carcinogenic process.

Cultured non-transformed cells can survive exclusively in anchorage-dependent conditions. When loss of cell-cell and/or cell-matrix interactions occurs, cell death ensues. This is termed anoikis, and resistance to anoikis is a common feature of cancer cells [4]. In addition to carcinogenesis, anoikis resistance also relates to cancer stem cell (CSC) like phenotypes, chemoresistance, and propensity to metastasize [5, 6, 7]. However, not all cancer cells are resistant to anoikis. We previously reported that some HGSOC cell lines do not attain anoikis resistance [8]. Several oncogenic signaling pathways are involved in resistance to anoikis. In HGSOC, anoikis resistance is related to phosphorylation of ERK1/2, p38MAPK, JNK and Src [5, 9, 10, 11].

A functional genomics screen is an effective method to identify genes that are truly responsible for specific functions or phenotypes among various genetic alterations that occur in cancer cells. The use of an shRNA library is one of the most effective research tools to carry out functional genomics screening [12]. Recently, novel tumor suppressor genes in colon cancer and breast cancer were identified through functional genomics screening using an shRNA library [13, 14]. There are several reports of functional genomics screens using shRNA libraries in ovarian cancer [15, 16, 17]. However, to our knowledge this is the first functional genomics screen to select shRNAs that enable ovarian cancer cells to grow in anchorage-free conditions. We chose to use soft agar colony formation assays since they have commonly been used for evaluating resistance to anoikis as well as for functional genomics screens [18, 19].

We analyzed the status of the identified genes in clinical samples. Our results suggest a novel approach to identify genes functionally responsible for malignant phenotypes of HGSOC and the various genetic alterations that occur in this disease.

## RESULTS

### Functional genomic screening

#### First shRNA library screening

**Figure 1:**
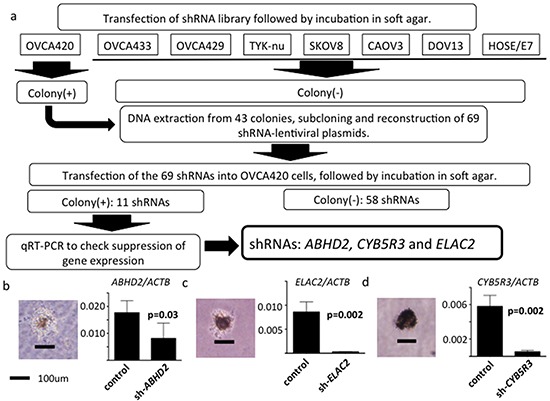
Schematic of functional genomics screens **a.** Seven human ovarian serous adenocarcinoma cell lines and an immortalized human ovarian surface cell line HOSE/E7, all of which do not grow in soft agar, were used. Following transfection of the shRNA library, only OVCA420 cells formed colonies in soft agar. 43 colonies were successfully expanded. shRNAs were amplified by PCR and we reconstructed 69 different shRNA plasmids. Out of the 69 shRNAs in OVCA420 cells, 11 again generated colonies in soft agar. We then measured mRNA expression of these 11 genes using RT-PCR. Of the 11 shRNAs, shRNAs directed against *ABHD2, CYB5R3* and *ELAC2* suppressed target gene mRNA expression. **b.** Left: shRNA-*ABHD2* transfected OVCA420 cell colony in soft agar. Black bar, 100 μm. Right: normalized *ABHD2* / *ACTB* mRNA expression analyzed by RT-PCR. (n=3, respectively) **c.** Left: shRNA-*ELAC2* transfected OVCA420 cell colony in soft agar. Right: normalized *ELAC2* / *ACTB* mRNA expression. **d.** Left: shRNA-*CYB5R3* transfected OVCA420 cell colony in soft agar. Right: normalized *CYB5R3* / *ACTB* expression.

Schematics of the functional genomics screens used are shown in Figure 1a.

We previously reported on seven serous ovarian cancer cell lines, including OVCA420, OVCA433, OVCA429, TYK-nu, SKOV8, CAOV3 and DOV13, that do not exhibit anchorage-independent cell proliferation [8]. These seven serous ovarian cancer cell lines and HOSE-E7 [20] were used in the first screening. We transduced the DECIPHER RNAi library Module (Cellecta, Mountain View, USA), a pRSI12-based backbone lentiviral shRNA library comprising ~80,000 plasmids targeting ~15,000 genes, into cells according to the manufacturer's instructions. Using the pRSI12 backbone control vector, we carefully repeated preliminary experiments to find optimal conditions for transduction of all kinds of shRNA constructs into cells. Using these conditions, we transduced an shRNA library into cells at a high multiplicity of infection (>0.5). Following 72 hours of selection with puromycin, 3.6×10^6^ stably transduced cells were suspended in 0.3% soft agar with 1x media for soft agar colony assays. On day 21, only OVCA420 cells (among the eight cell lines) formed anchorage independent colonies >100 μm following shRNA library transduction. They were selected under a microscope and individually expanded. Forty-three colonies were successfully expanded, from which we extracted DNA for subcloning.

### Subcloning and reconstruction of shRNA plasmids, followed by a second screening

We conducted a second screen using shRNAs selected in the first screen by reconstructing lentiviral plasmids. The shRNA target sequences were located between the *Cla*I and *Xba*I restriction sites in the pRSI12 lentiviral vector. We amplified shRNA target sequences by PCR from DNA extracted from the colonies that were grown in soft agar. The amplified PCR products were subcloned into the original pRSI12 lentiviral vector at the *Cla*I and *Xba*I sites, thus reconstructing pRSI12 shRNA lentiviral plasmids. Because multiple shRNA plasmids could be represented in one colony, we subcloned at least five clones per PCR amplicon. We identified 69 different shRNAs by Sanger sequencing, targeting 66 genes (Supplementary Table S1). In the second screen, we transfected the 69 reconstructed lentiviral shRNA plasmids into OVCA420 cells individually and performed soft agar colony formation assays. We repeated the second screen in triplicate, confirming reproducibility. On day 21, 11 shRNA plasmid-transduced OVCA420 cells formed colonies >100 μm in diameter (Supplementary Table S2) and thus were regarded as reproducible effects of these shRNAs. We then evaluated mRNA expression in the 11 different shRNA-transduced OVCA420 cells by RT-PCR to exclude off-target effects. We found that three different shRNA plasmids, whose target genes were *ABHD2, ELAC2* and *CYB5R3*, suppressed mRNA expression of their targets (p=0.03, p=0.0202, p=0.002, respectively, Figure 1b-1d).

### Expression and copy number alterations of *ABHD2, ELAC2* and *CYB5R3*

**Figure 2:**
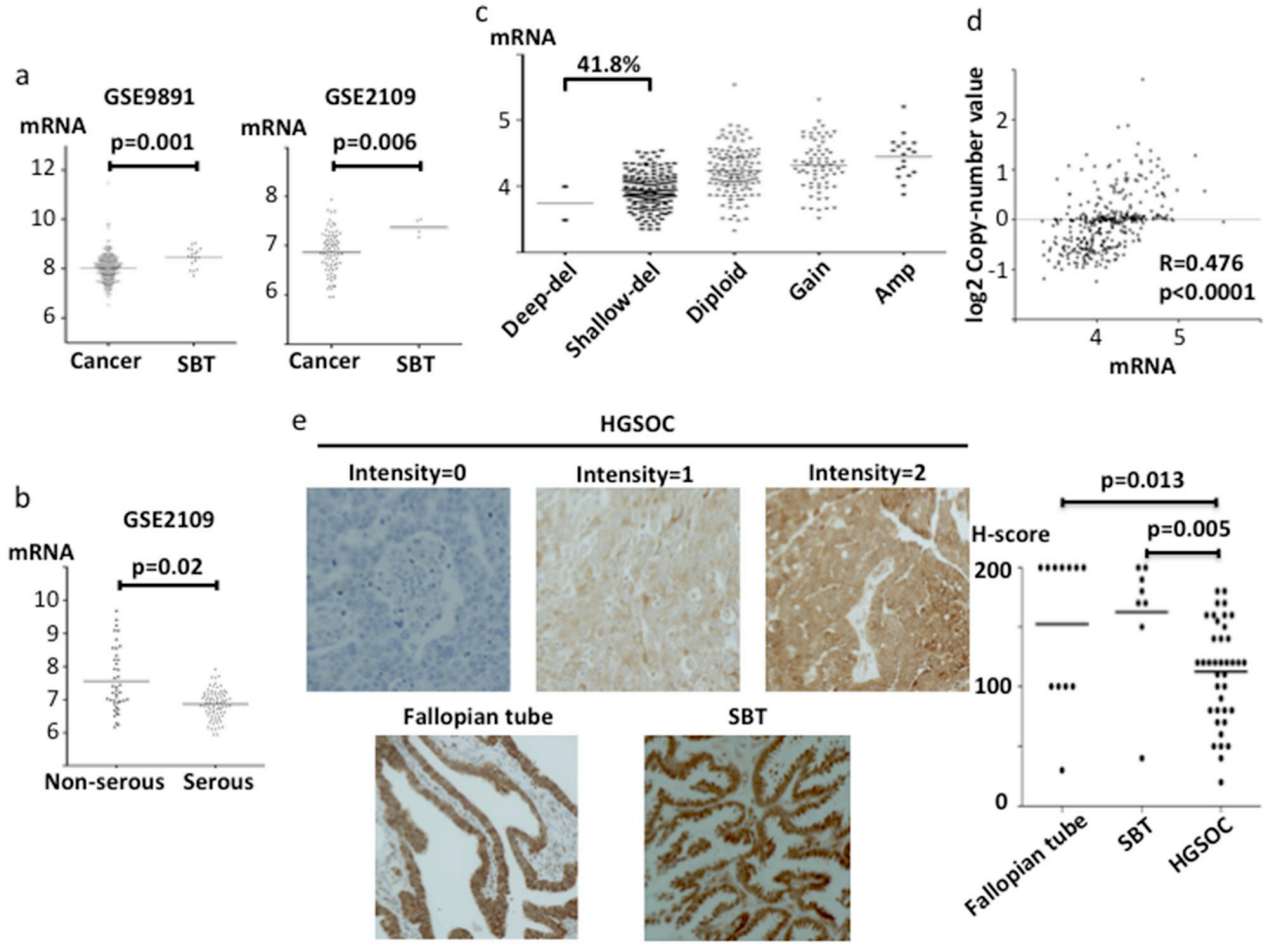
*ABHD2* mRNA, protein expression and copy number in clinical specimens mRNA expression was evaluated using log2 normalized values. **a.** Comparison of *ABHD2* mRNA expression between ovarian cancer tissues and serous borderline tumors (SBT) using gene expression microarray datasets GSE9891 and GSE2109. **b.** Comparison of *ABHD2* mRNA expression between serous adenocarcinoma and non-serous adenocarcinoma in microarray dataset GSE2109. **c.** Copy number alterations for *ABHD2* in TCGA samples. Del; deletion, Amp; Amplification. **d.** Correlation between *ABHD2* copy number and mRNA expression in TCGA specimens. **e.** Representative ABHD2 immunohistochemistry staining for HGSOC (intensity 0, 1 and 2), normal fallopian tube and SBT are shown. Comparison of H-scores among HGSOC, fallopian tube and SBT. The H-score is calculated as 2x the percentage of the most strongly stained area plus 1x the percentage of the most weakly stained area, imparting a total score ranging from 0 to 200.

We next investigated gene expression and potential copy number changes for *ABHD2, ELAC2* and *CYB5R3* in clinical samples. For *ABHD2*, mRNA expression was significantly lower in ovarian cancer than that in serous borderline tumors (SBT) (p=0.001; GSE9891, p=0.006; GSE2109, Figure 2a). In addition, among the ovarian cancers, expression was significantly lower in HGSOC than that in non-HGSOC (p=0.02; GSE2109, Figure 2b). Furthermore, loss of the *ABHD2* locus at 15q26.1 was frequent in HGSOC samples from the TCGA dataset (41.8%, Figure 2c). We found a positive correlation between copy number and mRNA expression (r=0.476, p<0.0001, Figure 2d). Additionally, we investigated ABHD2 protein expression in clinical samples by immunohistochemistry. Consistent with the observed reduction in mRNA, there was a statistically significant reduction in ABHD2 protein expression in HGSOC (n=36) as compared to SBT (n=8) and normal fallopian tube (n=11) (p=0.005 and p=0.013, respectively, Figure 2e).

**Figure 3:**
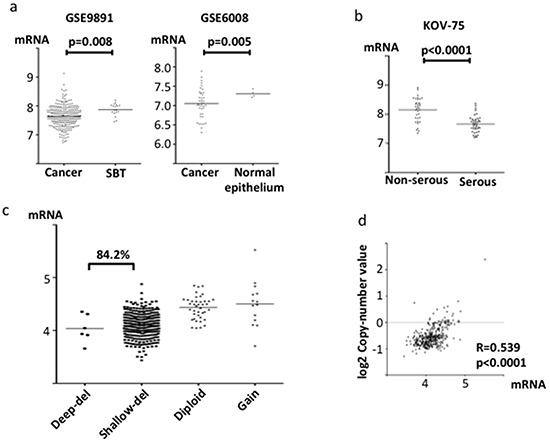
*ELAC2* mRNA expression and copy number in clinical specimens mRNA expression was evaluated using the log2 normalized values. **a.** Comparison of *ELAC2* mRNA expression between ovarian cancer tissue and SBT or normal ovarian epithelium from the gene expression microarray datasets GSE9891 and GSE6008. **b.** Comparison of *ELAC2* mRNA expression between serous adenocarcinoma and non-serous adenocarcinoma in microarray dataset KOV-75. **c.** Copy number alterations at *ELAC2* in TCGA specimens. **d.** Correlation between *ELAC2* copy number and mRNA expression in the TCGA dataset.

**Figure 4:**
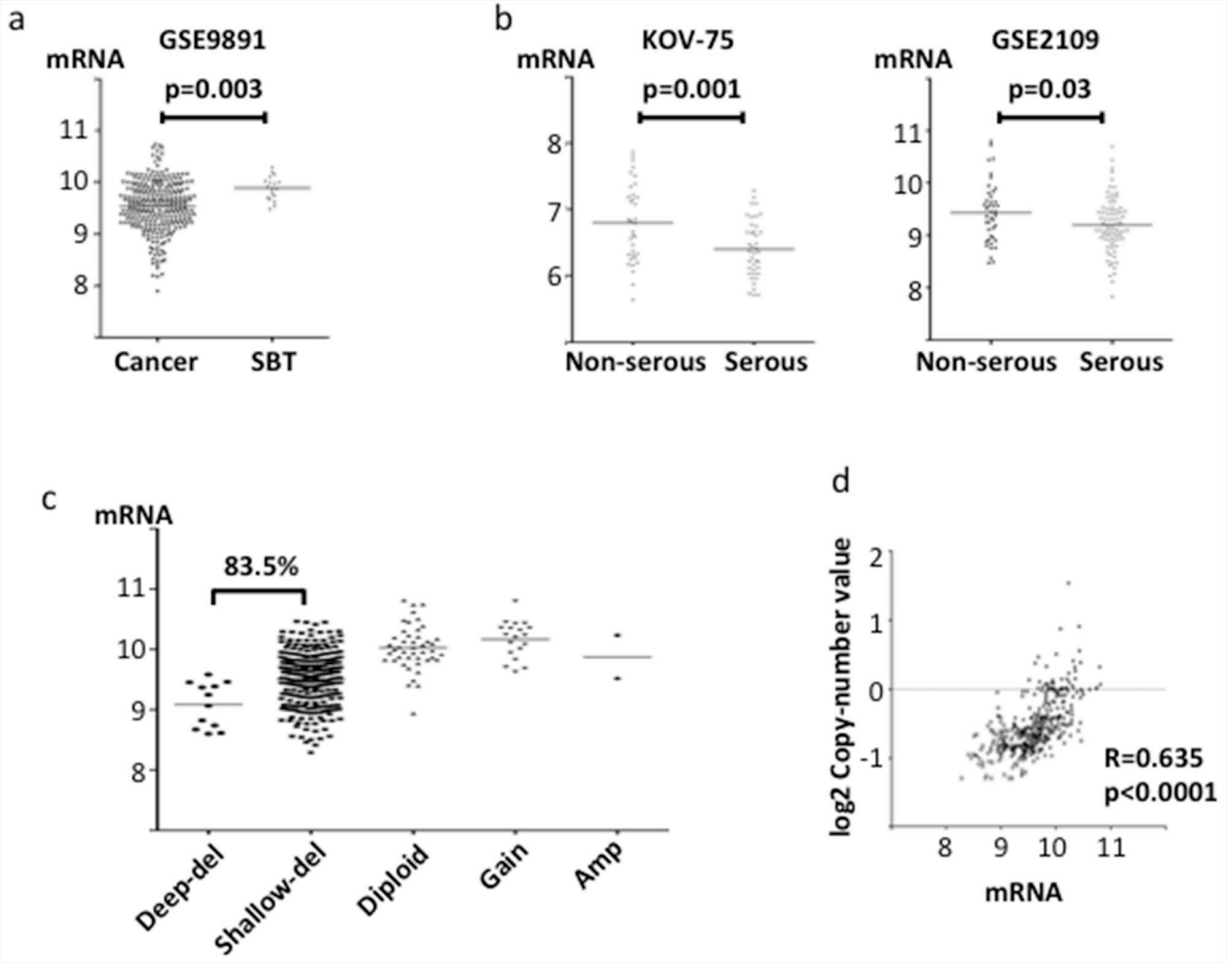
*CYB5R3* mRNA expression and copy number in clinical specimens mRNA expression was evaluated using the log2 normalized value. **a.** Comparison of *CYB5R3* mRNA expression between ovarian cancer tissue and SBT in the gene expression microarray dataset GSE9891. **b.** Comparison of *CYB5R3* mRNA expression between serous adenocarcinoma and non-serous adenocarcinoma in microarray datasets KOV-75 and GSE2109. **c.** Copy number alterations of *CYB5R3* in TCGA specimens. **d.** Correlation between *CYB5R3* copy number and mRNA expression in the TCGA dataset.

Like *ABHD2*, *ELAC2* and *CYB5R3* mRNA levels were significantly lower in ovarian cancer than in SBT (p=0.008 and 0.003, respectively, Figure 3a, Figure 4a). *ELAC2* expression in cancer was also lower than that in normal ovarian epithelium (p=0.005, Figure 3a). Comparing histologic subtypes of epithelial ovarian cancers, *ELAC2* and *CYB5R3* mRNA levels were significantly lower in HGSOC than in non-HGSOC (p<0.0001, p=0.03, respectively, Figure 3b, Figure 4b). In the TCGA dataset, genomic deletions at *ELAC2* (17p11.2) and at *CYB5R3* (22q13.2) were frequent in HGSOC (84.2% and 83.5%, respectively, Figure 3c, Figure 4c). Both showed positive correlations between mRNA levels and the copy number reported (*ELAC2*; r=0.539, p<0.0001, *CYB5R3*; r=0.485, p<0.0001, Figure 3d, Figure 4d).

Next, we analyzed promoter methylation and also looked for the presence of DNA mutations for the three genes identified from the HGSOC TCGA data. Except for two cases that had a missense mutation in *ABHD2*, there were no other mutations in the three genes (data not shown). *ELAC2* and *CYB5R3* were mostly unmethylated (Supplementary Figure S1a). *ABHD2* showed some evidence of methylation (Supplementary Figure S1a), but when we analyzed the extent of methylation in the promoter region of *ABHD2* in two ovarian cancer cell lines (HEYA8 and A2780) and eight HGSOC samples by bisulfite sequencing, we found that there were few methylated CpG dinucleotides in the promoter region. (Supplementary Figure S1b, S1c). Therefore, we concluded that promoter methylation and DNA mutations play a minimal role in the regulation of expression for these three genes in HGSOC.

In summary, altered *ABHD2*, *ELAC2* and *CYB5R3* mRNA expression was identified through our functional genomics screen and was significantly reduced in ovarian cancer, especially in HGSOC, relative to that in SBT or normal ovarian epithelium. In addition, these genes are located in loci that are frequently deleted in HGSOC. A positive correlation between mRNA expression levels and copy number was detected for all three genes.

### Validation of ABHD2 as a negative regulator of anoikis resistance

Among the three genes that we identified, mRNA expression levels of *ABHD2* were related to prognosis (described below). We therefore focused on the role of *ABHD2* in ovarian cancer.

**Figure 5:**
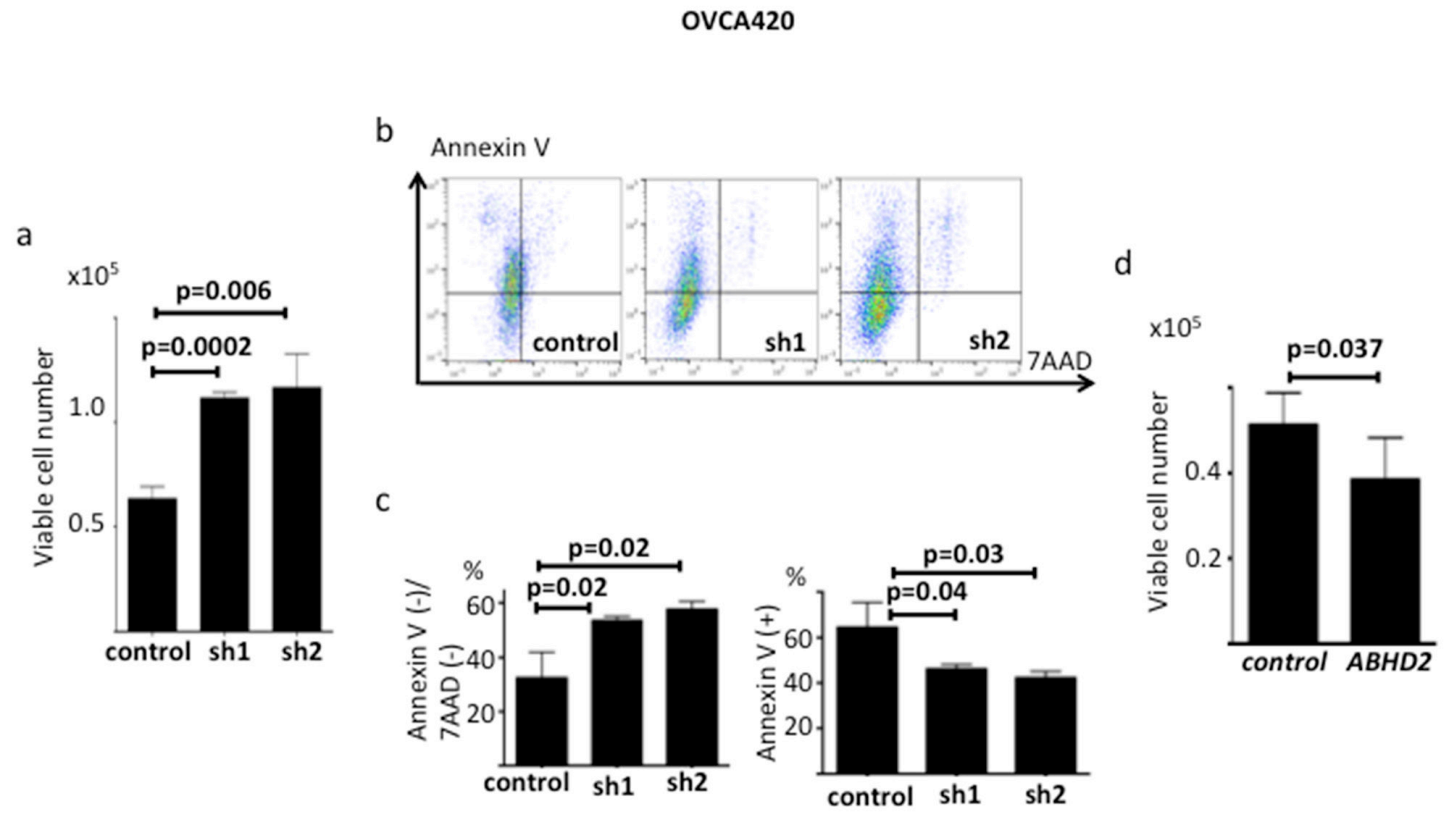
Functional validation of ABHD2 as a negative regulator of anoikis resistance in OVCA420 cells **a.** Number of viable control, sh1-OVCA420 and sh2-OVCA420 cells following incubation on ultra-low attachment plates (n=6). **b.** Representative data showing Annexin V/7-ADD staining following incubation on ultralow attachment plates. **c.** Comparison of the ratio of the Annexin V(−)/7-ADD(−) fraction (viable cells) and Annexin V(+) fraction (apoptotic cells) between control, sh1 and sh2 cells. **d.** Number of viable OVCA420-control and OVCA420-*ABHD2* cells following incubation on ultra-low attachment plates (n=6). Panels a-c: sh1; sh1-OVCA420, sh2; sh2-OVCA420, control; control-OVCA420; panel d: *control;* OVCA420-control, *ABHD2*; SKOV3-*ABHD2*.

We transduced *ABHD2-*specific shRNAs, different from those used for the functional screening, and non-silencing control shRNA plasmids into OVCA420 cells, and established sh1-OVCA420, sh2-OVCA420 and control-OVCA420, respectively (Supplementary Figure S2a). We confirmed that both *ABHD2* mRNA and protein expression were suppressed in sh1-OVCA420 and sh2-OVCA420 compared to those in control-OVCA420 by RT-PCR and Western blotting (Supplementary Figure S2a). To assess resistance to anoikis, we employed a more quantitative method than the colony formation assay. We cultured cells in ultra-low attachment dishes and counted viable cells after seven days. We found that both sh1-OVCA420 and sh2-OVCA420 cells had significantly enhanced survival relative to the control-OVCA420 cells (Figure 5a). Next we assessed the expression of AnnexinV, a marker of apoptosis, following incubation in ultra low attachment dishes for 24 hours. Expression of AnnexinV in sh1-*ABHD2*, sh2-*ABHD2* OVCA420 cells was significantly lower than that of control OVCA420 cells (Figure 5b, 5c). Therefore, suppression of *ABHD2* caused resistance to apoptosis under anchorage-free conditions, which has also been referred to as anoikis resistance. There was no significant difference in proliferation for the sh1-OVCA420, sh2-OVCA420 and control-OVCA420 cells grown in standard tissue culture dishes (Supplementary Figure S3a). We established ABHD2-overexpressing OVCA420 cells by transfecting the cells with an expression plasmid containing the open reading frame of *ABHD2* (Supplementary Figure S2b) (referred to hereafter as OVCA420-*ABHD2* cells) and assessed their resistance to anoikis. OVCA420-*ABHD2* cells had significantly lower resistance to anoikis relative to that of OVCA420 cells transfected with the vector control plasmid (OVCA420-control cells) (Figure 5d).

**Figure 6:**
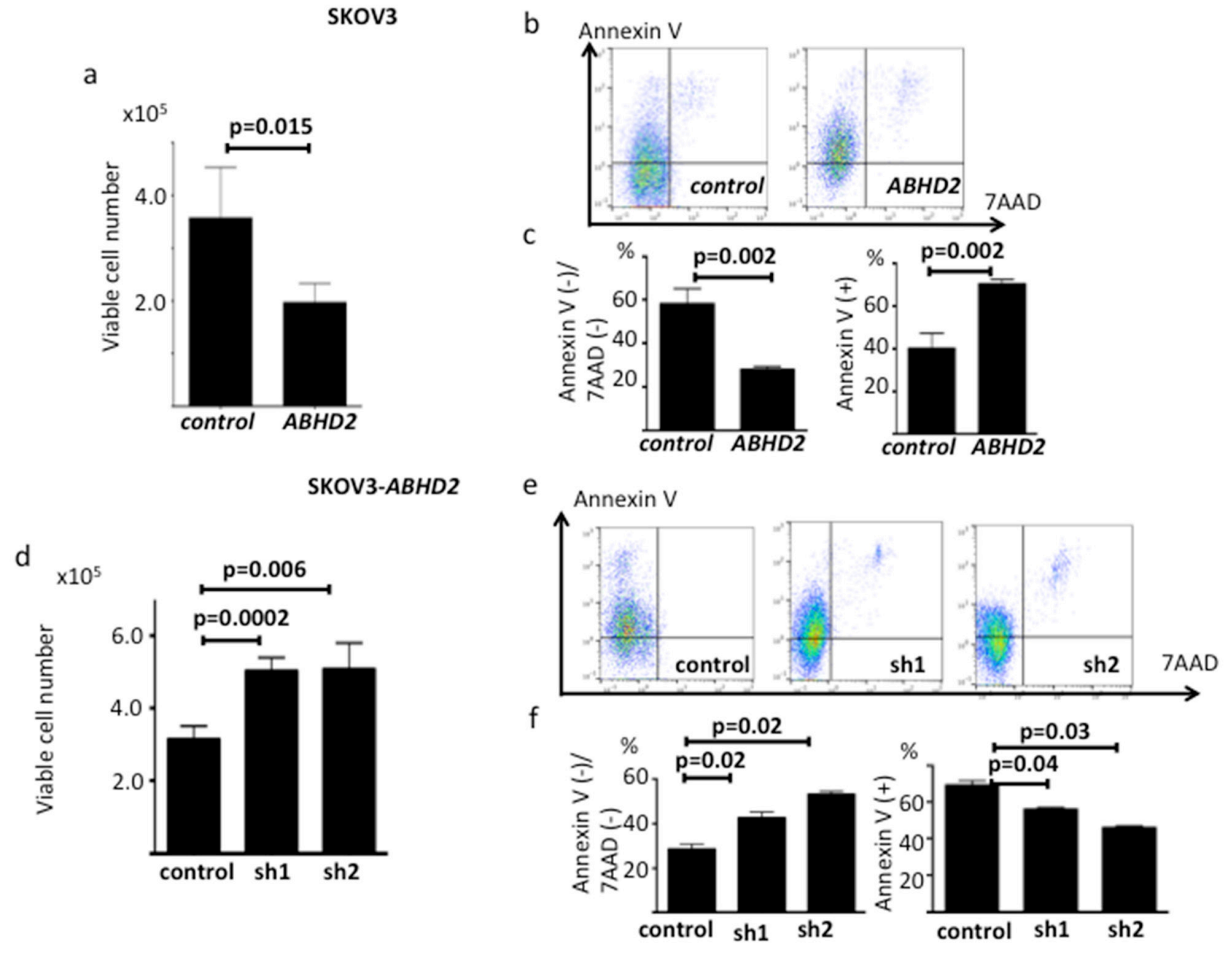
Functional validation of ABHD2 as a negative regulator of anoikis resistance in SKOV3 cells **a.** Number of viable SKOV3-control and SKOV3-*ABHD2* cells following incubation on ultra-low attachment plates (n=6). **b.** Representative data showing Annexin V/7-ADD staining following incubation on ultralow attachment plates. **c.** Comparison of the ratio of the Annexin V(−)/7-ADD(−) fraction and Annexin V(+) fraction between SKOV3-control and SKOV3-*ABHD2* cells. **d.** Number of viable control- SKOV3, sh1- SKOV3-*ABHD2* and sh2-SKOV3-*ABHD2* cells following incubation on ultra-low attachment plates (n=6). **e.** Representative data showing Annexin V/7-ADD staining following incubation on ultralow attachment plates for 48 hours. **f.** Comparison of the ratio of the Annexin V(−)/7-ADD(−) fraction and Annexin V(+) fraction among control, sh1-SKOV3-*ABHD2* and sh2-SKOV3-*ABHD2* cells. Panels a-c: *control*; SKOV3-control, *ABHD2*; SKOV3-*ABHD2*; panels d-f: control; control-SKOV3-*ABHD2,* sh1; sh1-SKOV3-*ABHD2,* sh2*;* sh2-SKOV3-*ABHD2*.

We next transduced SKOV3 cells with a plasmid containing the open reading frame of *ABHD2* or a vector-only control plasmid, establishing SKOV3-*ABHD2* cells and SKOV3-control cells, respectively (Supplementary Figure S2c). We confirmed elevated expression of ABHD2 mRNA and protein in SKOV3-*ABHD2* cells compared to SKOV3-control cells using RT-PCR and Western blotting (Supplementary Figure S2c). We assessed resistance to anoikis in the same way as described above, and found that SKOV3-*ABHD2* cells were less able to survive in an ultra-low attachment dish than were the SKOV3-control cells (Figure 6a). When we incubated SKOV3-*ABHD2* and control cells on ultra-low attachment dishes for 48 hours, AnnexinV expression was significantly higher in SKOV3-*ABHD2* cells relative to SKOV3-control cells (Figure 6b, 6c). In standard tissue culture dishes, there was no difference in proliferation between the SKOV3-*ABHD2* and SKOV3-control cells (Supplementary Figure S3b). Furthermore, we transfected SKOV3*-*ABHD2 cells with control shRNA, *ABHD2*-specific shRNA-1 or *ABHD2*-specific shRNA-2 to establish control-SKOV3-*ABHD2*, sh1-SKOV3-*ABHD2* and sh2-SKOV3-*ABHD2* cells (Supplementary Figure S2d). Anoikis resistance, assessed by AnnexinV expression following incubation on ultra-low attachment dishes, was significantly increased in sh1-SKOV3-*ABHD2* and sh2-SKOV3-*ABHD2* cells as compared to the control-SKOV3-*ABHD2* cells (Figure 6d, 6e, 6f).

We overexpressed or suppressed *ABHD2* expression in RMG1, an ovarian clear cell carcinoma cell line. Then we could not find significant difference among control, *ABHD2-*overexpressed and *ABHD2*-suppressed cells (in terms of anoikis resistance (data not shown.)

As a result, we could find that *ABHD2* expression regulated anoikis resistance of HGSOC.

### Analysis of intracellular signaling relevant to anoikis resistance

**Figure 7:**
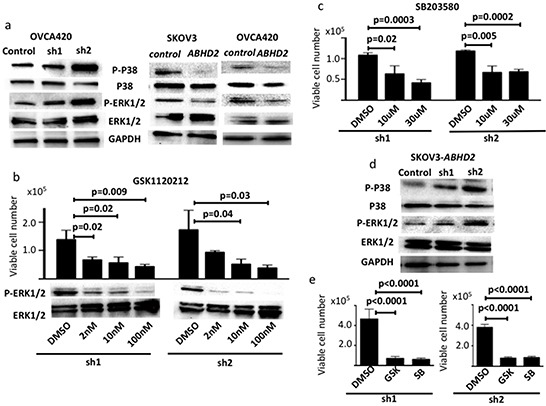
Regulation of ERK1/2 and p38MAPK pathways by ABHD2 All experiments were performed in triplicate. **a.** Phosphorylated p38 (P-P38) and phosphorylated ERK1/2 (P-ERK1/2) increased following knockdown of *ABHD2* (sh1 and sh2) in OVCA420 cells. On the contrary, P-P38 and P-ERK1/2 decreased following overexpression of *ABHD2* in SKOV3 and OVCA420 cells. **b.** Resistance of OVCA420 cells to anoikis on ultra-low attachment dishes was inhibited by GSK1120212, a specific inhibitor of the ERK1/2 pathway. Reduction of P-ERK1/2 following treatment with GSK1120212 was confirmed by Western blotting. DMSO, vehicle control. Cells were treated with differing doses of GSK1120212 as indicated. **c.** Resistance of OVCA420 cells to anoikis was inhibited following treatment with SB203580, a specific inhibitor of the the p38MAPK pathway. **d.** Levels of P-P38 and P-ERK1/2 increased following knockdown of *ABHD2* (sh1 and sh2) in SKOV3-*ABHD2* cells. **e.** Resistance to anoikis in sh1-SKOV3-*ABHD2* and sh2-SKOV3-*ABHD2* cells was inhibited following treatment with 100nM GSK1120212 (GSK) and 30μM SB203580 (SB).”

We examined phosphorylation of ERK1/2, p38MAPK, JNK and Src by Western blotting since they have been reported as relevant to anoikis resistance in ovarian cancer [5, 9, 10, 11]. Phosphorylation of ERK1/2 and p38MAPK was increased in sh1-OVCA420 and sh2-OVCA420 cells compared to control-OVCA420 (Figure 7a). Consistent with these results, phosphorylation was decreased in both SKOV3-*ABHD2* and OVCA420-*ABHD2* cells as compared to SKOV3-control and OVCA420-control cells, respectively (Figure 7a). We did not detect any difference in phosphorylation status for JNK and Src (data not shown).

We next assessed the effects of specific inhibitors of the ERK (GSK1120212) or p38MAPK (SB203580) pathways [21, 22]. Treatment with both GSK1120212 and SB203580 decreased anoikis resistance that was primed by suppression of *ABHD2* in OVCA420 cells (Figure 7b, 7c). Phosphorylation of ERK1/2 and p38MAPK was increased in sh1-SKOV3-*ABHD2* and sh2-SKOV3-*ABHD2* cells as compared to control-SKOV3-*ABHD2* cells (Figure 7d). Following incubation in ultra-low attachment dishes, the number of viable sh1-SKOV3-*ABHD2* and sh2-SKOV3-*ABHD2* cells was significantly decreased when the cells were treated with GSK1120212, a specific inhibitor of the ERK1/2 pathway, or SB203580, a specific inhibitor of the p38MAPK pathway (Figure 7e). These results indicate suppression of *ABHD2* enhances anoikis resistance in ovarian cancer via activation of both ERK1/2 and p38MAPK pathways.

### Association of ABHD2 expression and clinicopathological factors in HGSOC

**Figure 8:**
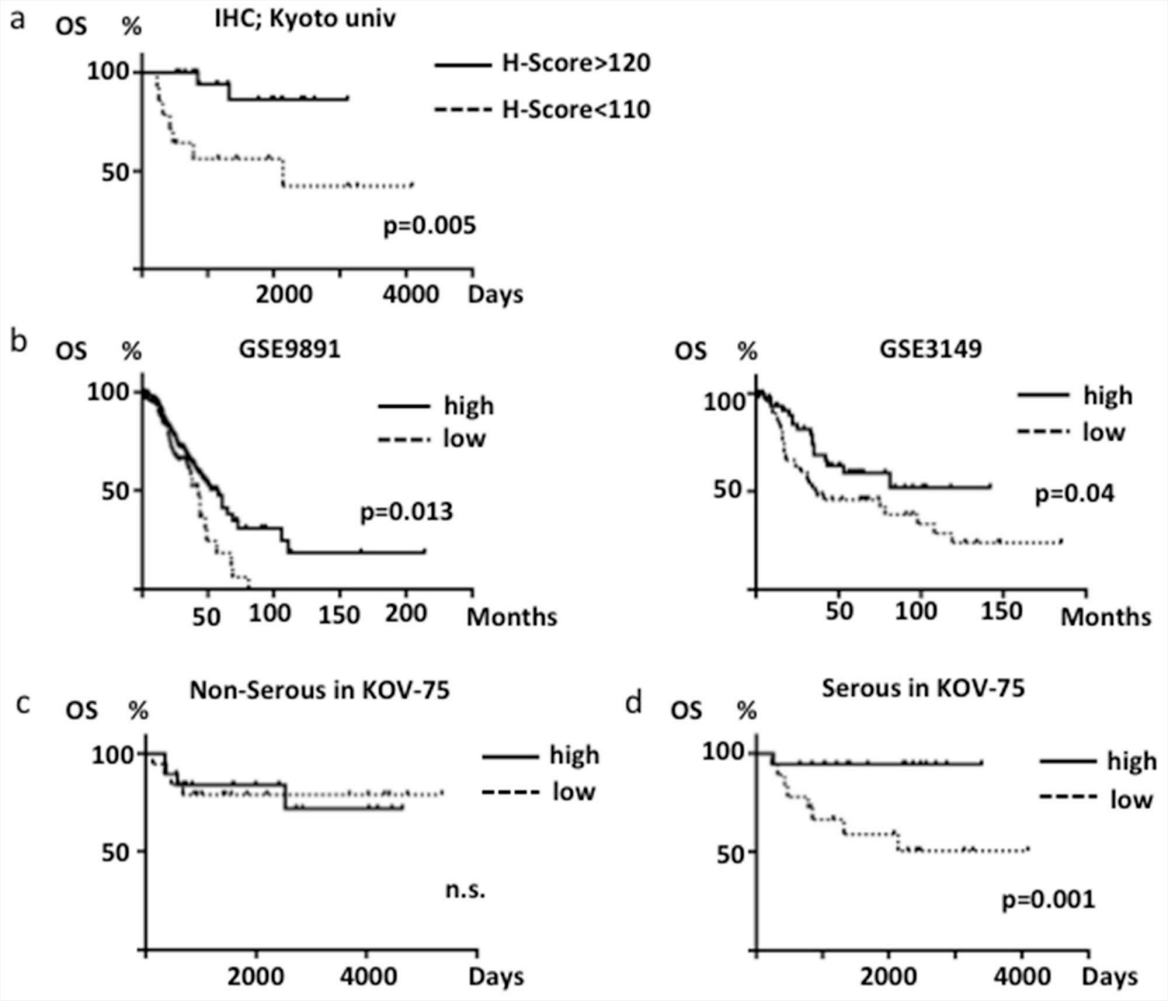
Overall survival of ovarian cancer patients **a.** Differences in survival based on ABHD2 immunohistochemical scores (H-score) in HGSOC. **b.** Differences in survival based on *ABHD2* mRNA expression in GSE9891 (n=285, mostly HGSOC) and GSE3149 (n=146, mostly HGSOC) datasets. Samples were divided into high (greater than the median value) and low (less than the median) expression cases. **c.** Analysis of HGSOC patients (n=36) from KOV-75 based on *ABHD2* mRNA expression. **d.** Analysis of non-HGSOC patients (n=39) from KOV-75 based on *ABHD2* mRNA expression. n.s., not significant.

We investigated the relationship between ABHD2 protein expression and clinicopathological factors in HGSOC, including patient age, FIGO stage, ascites cytology, lympho-vascular invasion (LVSI), lymph node metastasis, platinum resistance and prognosis. We regarded patients who recurred within one year from completion of treatment as platinum-resistant cases as has been previously reported [23]. As a result, we found there were significantly more platinum-resistant patients in the low-ABHD2 group than in the high-ABHD2 group (p=0.013, Table 1). The prognosis of the low-ABHD2 group patients was significantly worse as compared to that of the high group patients (p=0.005, Figure 8a), although it was not an independent prognostic factor. In accordance with this result, in two expression microarray datasets of ovarian cancer consisting mostly of HGSOC (GSE9891, GSE3149), the prognosis of the low *ABHD2* expression group was significantly worse as compared to that of the high *ABHD2* expression group (p=0.013, p=0.04, respectively, Figure 8b).

**Table 1:** ABHD2 expression levels and clinicopathological factors

H-score	<110 (n=15)	>120 (n=21)	p-value
Age	55.8	56.9	0.89
FIGO stage			
1,2	4	5	
3,4	11	16	0.69
Ascites			
+	12	12	
-	3	9	0.28
Clinical platinum sensitivity			
Sensitive	9	20	
Resistance	6	1	0.013
LVSI			
+	11	9	
-	4	12	0.096
Lymph node metastasis			
+	8	6	
-	5	14	0.15
(unknown)	2	1	

In order to examine if differences between histological subtype affect the difference in prognosis, we divided the KOV-75 gene expression microarray specimens into HGSOC (n=36) and non-HGSOC (n=39). There were no significant differences in prognosis based on expression levels of *ELAC2* or *CYB5R3* in HGSOC versus non-HGSOC (data not shown). Similarly, there was no significant difference in prognoses based on expression levels of *ABHD2* in the non-HGSOC cases (Figure 8c). However, the prognosis of the low-*ABHD2* expression group was significantly worse in HGSOC (p=0.001, Figure 8d). Thus, low expression of *ABHD2* is significantly related to platinum-resistance and a poor prognosis for HGSOC. We also analyzed expression of *ELAC2* or *CYB5R3* and if expression is related to ovarian cancer prognosis, but there was no significant relationship for either gene (data not shown).

### Alteration of platinum sensitivity by decreased ABHD2 expression *in vitro*

**Figure 9:**
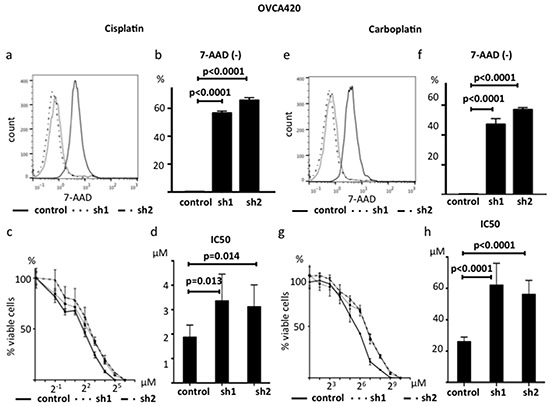
Suppression of *ABHD2* causes platinum resistance **a.** Representative data showing 7-ADD staining following 24 hour incubation with 10 μM cisplatin. **b.** The ratio of the 7-AAD negative live OVCA420 cells markedly increased following suppression of *ABHD2* (sh1 and sh2) as compared to the control after 24 hour incubation with 10 μM cisplatin (n=3). **c.** Dose-response curves following incubation of OVCA420 cells with the indicated concentrations of cisplatin for 72 hours (n=6). **d.** Cisplatin IC50 values increased following suppression of *ABHD2* in OVCA420 cells. **e.** Representative data showing 7-ADD staining following 24 hour incubation with 100 μM Carboplatin. **f.** The ratio of 7-AAD negative live OVCA420 cells markedly increased following suppression of *ABHD2* (sh1 and sh2) as compared to the control following a 24 hour incubation with 100 μM carboplatin (n=3).

Because decreased expression of *ABHD2* is related to platinum resistance in clinical samples, we investigated whether suppression of *ABHD2* could confer resistance to platinum *in vitro*. We cultured sh1-*ABHD2*, sh2-*ABHD2* and control- OVCA420 cells with 10 μM CDDP for 24 hours, followed by 7AAD staining to detect dead cells. The ratio of 7AAD-negative cells was significantly increased in sh1-*ABHD2* and sh2-*ABHD2* cells as compared to that of control cells (p<0.0001, Figure 9a, 9b). We determined the CDDP IC50 values at 72 hours and found that sh1-*ABHD2* and sh2-*ABHD2* cells exhibited significantly higher IC50 values than that of control cells (p=0.013, p=0.014, respectively, Figure 9c, 9d). Additionally, we examined sensitivity to Carboplatin (CBDCA) through identification of dead cells and comparison of IC50 values. The results were consistent with those of CDDP (p<0.0001, Figure 9e, 9f, p<0.0001, Figure 9g, 9h). Furthermore, we used another HGSOC cell line, CAOV3, and investigated whether suppression of ABHD2 could also confer resistance to platinum. We established sh1-CAOV3 cells and control cells in the same manner, and found that sh1-CAOV3 cells were significantly more resistant to CDDP (Supplementary Figure S4a, S4b). Therefore, suppression of *ABHD2* confers resistance to platinum *in vitro*.

### Side population and *ABHD2* expression

The side population (SP) cell fraction refers to the small population of cells that have a high capacity to efflux Hoechst dye 333432. Because an increase in the SP fraction is reported to be associated with resistance to anoikis [24], we investigated if the SP fraction is altered by suppression of *ABHD2* expression in OVCA420 cells. We found that the SP fraction of sh1-*ABHD2* and sh2-*ABHD2* cells was significantly higher than that of control cells (p<0.001, p<0.0001, respectively, Supplementary Figure S5).

## DISCUSSION

In this research, we performed a functional genomics screen using an shRNA library to search for drivers of anoikis resistance in HGSOC. This is the first report that refers to a positive screen about anoikis resistance, an unique function of HGSOC, using a shRNA library. Consequently, we successfully identified three genes, *ABHD2*, *ELAC2* and *CYB5R3* whose downregulation increased resistance to anoikis (Figure 1). mRNA expression from these three genes was lower in ovarian cancer tissue, especially in HGSOC relative to SBT or normal ovarian epithelium (Figure 2, 3, 4). Also, we found that loss of the chromosomal regions where these three genes are located occurred frequently in HGSOC (Figure 2, 3, 4). Thus, our functional genomics screen was useful in identifying driver changes involved in malignant phenotypes of HGSOC among the numerous copy number alterations that are characteristic of this disease. Similar to our results, the *REST* gene, identified as a tumor suppressor by using an shRNA library, is in a frequently deleted chromosomal location in colon cancer [13]. We found that DNA methylation and mutation were not related to downregulation of the three genes identified in HGSOC. These results are compatible with the prior findings that HGSOC is characterized by ubiquitous mutation of *TP53* and extensive copy number alterations, rather than other driver mutations and DNA methylation [25].

The immortalized human ovarian epithelial cell line HOSE/E7, established by transfection of hTERT and E7, does not form colonies in soft agar [21]. We found that shRNA library-transduced HOSE/E7 cells were also unable to form colonies in soft agar (Figure 1a).

Moreover, among the HGSOC cell lines that do not form colonies in soft agar, only OVCA420 formed colonies after transfection of the shRNA library. The publicly available microarray data [8] indicated that OVCA420 had higher *ABHD2* expression than the other cell lines (data not shown). This may be one reason that suppression of *ABHD2* expression caused resistance to anoikis only in the OVCA420 cells. Expression of *ELAC2* and *CYB5R3*, however, did not differ between OVCA420 and the other cell lines (data not shown). Therefore, the biological differences between OVCA420 and the other cell lines remain to be elucidated.

Among the three genes that we identified, we further analyzed *ABHD2* because low expression is a poor prognostic factor for HGSOC and there was an available antibody. *ABHD2* is a member of the alpha/beta hydrolase family. It was originally identified as a gene whose expression is suppressed in pulmonary emphysema [26]. Defects in *ABHD2* cause an increase of smooth muscle cell migration and of intimal hyperplasia. *ABHD2* deficient mice develop spontaneous gradual progression of emphysema [27, 28]. There are no prior reports of a relationship between *ABHD2* and cancer. The intimal cell hyperplasia caused by suppression of *ABHD2* might be related to apoptosis resistance observed in cancer cells. Suppression of another alpha/beta hydrolase member, *ABHD4,* was found to increase resistance to anoikis in RWPE-1 prostate cancer cells [29]. In our study, we found that suppression of *ABHD2* increases anoikis resistance through the ERK1/2 and p38MAPK pathways in HGSOC (Figure 7). How *ABHD2* affects changes in the ERK1/2 and p38MAPK pathways remains to be determined.

Intriguingly, we found a significant correlation between low expression of *ABHD2* and platinum-resistance in our clinical data analysis (Table 1). Further, suppression of *ABHD2* caused platinum resistance *in vitro* (Figure 9, Supplementary Figure S4). A correlation between anoikis resistance and platinum resistance was previously reported in HGSOC [30]. Moreover, it was also reported that ERK1/2 and p38MAPK pathway activation causes platinum resistance [31, 32]. It is also known that resistance to anoikis is a characteristics of cancer stem cells (CSC), and CSC have high drug efflux potential, thus inherent platinum resistance [6]. In our study, suppression of *ABHD2* increased the SP fraction of OVCA420 cells, a characteristic feature of CSC [33, 34, 35] (Supplementary Figure S5). The increase in colony formation in soft agar may indeed reflect an increase in stemness characteristics, but to demonstrate this would require a great deal of further work that is beyond the scope of the present manuscript. It was shown previously that suppression of *MKP3*, *DDB2* and *DOK2* increased resistance to anoikis, chemoresistance and enhanced the CSC phenotype of HGSOC [36, 37, 38]. Therefore, our results are consistent with those findings. Although the detailed molecular mechanisms by which suppression of *ABHD2* is involved in platinum-resistance remain unclear, our findings could be useful for development of a biomarker for individualized treatment of HGSOC.

In conclusion, we found that suppression of *ABHD2*, *ELAC2* or *CYB5R3* caused resistance to anoikis through a functional genomics screen. Loss of copy number of the loci containing these three genes was frequent in HGSOC, and as such may contribute to the HGSOC carcinogenic process. Suppression of *ABHD2* increased not only anoikis resistance, but also platinum-resistance. Our studies provide support for future research exploring previously undiscovered factors relevant to malignant phenotypes of HGSOC.

## MATERIALS AND METHODS

### Cell lines and reagents

We maintained human serous ovarian cancer cell lines including OVCA420, OVCA433, OVCA429, TYK-nu, SKOV8, CAOV3, DOV13, HEYA8, A2780 and SKOV3 [8], in RPMI 1640 (Nacalai Tesque, Kyoto, Japan) supplemented with 10% fetal bovine serum (FBS) and 100 IU/ml penicillin and 100 μg/ml streptomycin (Nacali Tesque). The immortalized human ovarian surface epithelial cell line, HOSE-E7 [20], kindly provided by Dr. Katabuchi at Kumamoto University, was cultured in DMEM/F12 (Thermo Fisher Scientific, Waltham, USA) supplemented with 10% FBS and 100 IU/ml penicillin and 100 μg/ml streptomycin (Nacalai Tesque). 293FT cells were purchased from Thermo Fisher Scientific, and cultured in DMEM (Thermo Fisher Scientific) supplemented with 10% FBS and 100 IU/ml penicillin and 100 μg/ml streptomycin. All cells were seeded into Cellstars® tissue culture plates (Greiner, Frickenhausen, Germany) in a humidified incubator containing 5% CO_2_ at 37°C. All cell lines were regularly tested for mycoplasma.

As an inhibitor of the p38 MAPK pathway, we used SB203580 (Sigma-Aldrich, St.Louis, USA). To inhibit the ERK1/2 pathway, we used GSK1120212 (Sigma-Aldrich).

### Soft agar colony formation assay

Soft agar colony formation assays were performed as previously described [39]. Briefly, the lower layer consisted of 1x media (RPMI1640 or DMEM/F12 with 10% FBS) and 0.6% low melting point agarose. Plates were chilled at room temperature until the agarose was solidified. The upper layer consisted of cells suspended in 1x media and 0.3% low melting point agarose. We added 1x medium to the top of the upper layer twice weekly. On day 21, we counted colonies that had attained a diameter of >100 μm.

### First shRNA library screening

We transfected the DECIPHER RNAi library Module (Cellecta, Mountain View, USA), a pRSI12 backbone lentiviral shRNA library comprising ~80 000 plasmids targeting ~15 000 genes into cells according to the manufacturer's instructions. In order to generate lentiviruses, we used Lipofectamin 2000 (Thermo Fisher Scientific) and Virapower (Thermo Fisher Scientific), and used 293FT cells as the packaging cells to generate the viral supernatant. Following 72 hours of selection with puromycin (Thermo Fisher Scientific), 3.6×10^6^ stably transduced cells were suspended in 0.3% soft agar with 1x media for soft agar colony assays as described above. In the first screening, we used twelve 10 cm dishes per cell line (3.0 × 10^5^ cells per dish). On day 21, colonies >100 μm were picked using 200 μl pipette tips (Nippon Genetics, Tokyo, Japan) under a microscope. Each colony was seeded into one well of a 96-well plate with culture medium and expanded until about 80% confluent in one well of a 24-well dish. We then extracted DNA using the DNeasy Blood and Tissue Kit (Qiagen, Venlo, Nederland) for subcloning.

### Subcloning and reconstruction of shRNA plasmids, followed by the second screening

In the pRSI12 lentiviral vector, an shRNA target sequence is located between *Cla*I and *Xba*I sites. We amplified shRNA target sequences of DNA extracted from the colonies grown in soft agar by PCR using the primers listed in Supplementary Table S3. Cycling parameters were 98°C for 5 minutes, followed by 30 cycles of 98°C for 10 seconds, 63°C for 10 seconds and 72°C for 30 seconds, followed by 72°C for 10 minutes. All the primers used in this study were purchased from Greiner. The amplified PCR products were subcloned into the original pRSI12 lentiviral vector at the *Cla*I and *Xba*I sites using the InFusion HD cloning Kit (Takara Bio, Otsu, Japan) according to the manufacturer's protocol, thus reconstructing pRSI12 shRNA lentiviral plasmids that were identical to those included in the original shRNA library. To identify the cloned shRNAs, we read the sense and anti-sense sequences by Sanger sequencing (3130xl Genetic Analyzer) (Thermo Fisher Scientific) using primers listed in Supplementary Table S3. In the second screening with the soft agar colony formation assay, we seeded 2.0×10^4^ cells per well in 6-well dishes.

### RNA extraction and real time quantitative PCR

Total RNA was extracted using the RNeasy® Mini Kit (Qiagen). RNA reverse transcription was performed using the Transcriptor High Fidelity cDNA Synthesis Kit (Roche, Basel. Switzerland) according to the manufacturer's instructions. Quantitative reverse transcriptase (RT)-PCR reactions of genes potentially targeted by shRNAs and Beta-Actin (*ACTB*) were performed using the Light Cycler 480-II (Roche) and a Mono Color Hydrolysis Universal Probe System (Roche). Primer sets were designed using the Universal Probe Library Assay Design Center (https://www.roche-applied-science.com/sis/rtpcr) and purchased from Greiner. Primer sets are listed in Supplementary Table S1. Cycling parameters were 95°C for 10 minutes followed by 45 cycles of 95°C for 10 seconds, 60°C for 30 seconds and 72°C for 1 second, followed by 40°C for 30 seconds. Relative mRNA expression levels were determined according to the ΔΔCt method, and values were normalized to *ACTB* expression.

### Bisulfite sequencing of cloned alleles

Methylation was analyzed by bisulfite PCR sequencing of cloned alleles. We extracted DNA from two ovarian cancer cell lines, A2780 and HEYA8, and from HGSOC tissue of eight patients using the DNeasy Blood and Tissue Kit (Qiagen). Two μg DNA was modified with sodium bisulfite treatment using the Bisulfite Kit (Qiagen) according to the manufacturer's protocol. We designed forward and reverse primers (Supplementary Table S1) that were used to amplify bisulfite modified DNA across the *ABHD2* promoter region. PCR products were purified using the QIAquick PCR Purification Kit (Qiagen), cloned into the TOPO-TA vector (Thermo Fisher Scientific) and sequenced following the manufacturer's standard protocol. Between 13 and 20 clones were sequenced for each sample.

### Stable knockdown and overexpression of *ABHD2*

TRC lentiviral *ABHD2*-specific shRNAs (GE Healthcare Life Science, Backinghamshire, UK) were transfected into OVCA420 cells as recommended by the manufacturer. Stably transfected cells were selected with puromycin (Thermo Fisher Scientific). We used two kinds of shRNAs whose target sequences were different from that of the sequence within the library of shRNAs. Target sequences were as follows; Sh1:ATGAGGAAGTTGTAGAACCGC, Sh2: TTACGCTCCCATTGGCAAATG. We also transfected non-silencing control TRC lentiviral shRNA plasmid into cells in the same manner.

We purchased precision LentiORF *ABHD2* plasmids (GE Healthcare Life Science), and transfected SKOV3 cells as recommended by the manufacturer. Stably transfected cells were selected with blasticidin (10ug/ml, Thermo Fisher Scientific). We also transfected control Precision LentiORF in the same manner.

### Western blot

Western blot was performed as previously reported [40]. Primary antibodies were anti-ABHD2 rabbit polyclonal antibody (1:250, Abgent AP13083c, San Diego, USA), anti-phospho ERK1/2 rabbit monoclonal antibody (1:1,000, Cell Signal Technology, 197G2, Danver, JAPAN), anti-ERK1/2 rabbit monoclonal antibody (1:1000, Cell Signal Technology, 137F5), anti-phospho p38 MAPK rabbit monoclonal antibody (1:1000, Cell Signal Technology, (D13E1)XP) anti-p38MAPK rabbit monoclonal antibody (1:1000, Cell Signal Technology, (D3F9)XP) and anti-GAPDH mouse monoclonal antibody (1:1000, Abcam, ab8245, Cambridge, UK). After washing in tris-buffered saline (TBS)-T, the blots were incubated with the appropriate peroxidase-coupled secondary antibody (1:2000; Anti-rabbit HRP or anti-mouse HRP, GE Healthcare Life Science). Specific proteins were detected using ECL Plus Western Blotting Reagent (GE Healthcare Life Science). The bands were visualized using Molecular Imager Gel DocTMXR+ and ChemiDocTMXRS+ Systems with Image Lab 2.0 software (Bio-Rad).

### Anoikis assays

Cells (1.0×10^5^ per well) were plated onto ultralow attachment 6-well plates (Corning, NY, USA) in 1x media to prohibit attachment. After incubation for 7 days, we collected cells and incubated them in trypsin-EDTA (0.25%) (Thermo Fisher Scientific) and Accumax (Innovative cell Technologies, San Diego, USA) for cell dissociation. We counted the total number of viable cells using a hemocytometer. Experiments were repeated in triplicate. Dead cells were excluded by trypan blue staining.

### Apoptosis assays

Cells were cultured in ultralow attachment plates (Corning) for 24 (OVCA420) or 48 (SKOV3) hours. The fraction of apoptotic cells was analyzed by MACSquant (MACSQuant) (Milteney Biotec, Cologne, Germany) following staining with APC-labeled annexin V and 7-Amino-actinomycin D (7AAD) (Becton, Dickinson and Company; BD, Franklin Lakes, USA) according to the manufacturer's protocol. Experiments were repeated at least three times.

### Cell proliferation assay

Cells were seeded into 96-well tissue culture plates at 2×10^3^ cells per well. At 8, 24, 48 and 72 hours cell viability was determined using the WST-1 assay kit (Premix WST-1®, Takara Bio) following the manufacturer's instructions. The absorbance values at 450nm were normalized by values at 8 hours following subtraction of values obtained from vacant wells.

### Platinum sensitivity assay

Cells were plated into 96-well plates at 2000 cells (OVCA420) or 3000 cells (CAOV3) per well. 24 hours later, the culture medium was replaced by fresh medium containing various concentrations of cisplatin (Nippon Kayaku, Kyoto, Japan) or carboplatin (Bristol Myers Squibb, NY, USA) for 72 hours. The percentage of viable cells was then examined using the WST-1 assay kit following the manufacturer's instructions. All cytotoxicity data shown are the means of at least three independent experiments.

7AAD (BD) staining was also performed to evaluate cytotoxicity. Following incubation with 10 μM CDDP or 100 μM CBDCA for 24 hours, cells were collected and stained with 2 μg/mL 7AAD. Dead cells were detected by flow cytometry (MACSQuant) following the manufacturer's instructions (BD).

### Patients and tissues

Ovarian tumor specimens and clinicopathological information were obtained from 36 patients with HGSOC, 8 patients with serous borderline tumor (SBT) and normal fallopian tube from 11 women, all of whom underwent primary surgery at Kyoto University Hospital between 1998 and 2014. All samples were obtained at the time of initial surgery without prior chemotherapy. Patient characteristics are listed in Supplementary Table S4. All tissue specimens were collected after obtaining written consent from patients with the approval of the Facility Ethical Committee, with approval number G288 (Nov 29^th^, 2013). All samples were fixed in 10% buffered formalin, embedded in paraffin, and sectioned.

### Immunohistochemical staining

Immunohistochemical staining was done using the streptavidin-biotin peroxidase complex method as previously reported [40]. Slides were incubated with a rabbit anti-ABHD2 antibody (Abgent) at a 1:40 dilution overnight at 4°C, followed by a one-hour incubation with biotinylated goat anti-rabbit secondary antibodies (Nichirei, Tokyo, Japan) at room temperature. Two examiners (KY and NM) independently evaluated the staining in a blinded fashion. We used the H-score to take staining intensity and proportion into consideration [41]. The score was assigned by a formula, i.e. 2 x percentage of a strongly stained area, 1 x percentage of a weakly stained area, imparting a total score ranging from 0 to 200. Staining intensity was assigned as weak, moderate or strong.

### Gene expression microarray datasets and copy number dataset

Microarray datasets were obtained from the Gene Expression Omnibus website (http://www.ncbi.nlm.nih.gov/gro). We used five gene expression microarray datasets (GSE2109, GSE6008, GSE3149, GSE9891, GSE55512 and GSE39204) to analyze differences in gene expression between ovarian cancer, ovarian borderline tumors and normal ovarian surface epithelium. Data from GSE55512 and GSE39204, both from our institution, were combined and termed KOV-75 [42, 43]. The cBioPortal database (http://www.cbioportal.org/public-portal/) was used to analyze copy number alterations and methylation status in HGSOC samples in The Cancer Genome Atlas (TCGA, 2011).

### Side population (SP) analysis

Cells were detached by trypsinization, centrifuged and resuspended in tissue culture medium containing 10% serum at a concentration of 1×10^6^ cells/mL.

At the end of the incubation, the cells were centrifuged and resuspended in cold PBS with 2% serum. The side population (SP) of cells was analyzed using a BD FACS AriaII system (BD) as previously reported [24]. The cells were labeled with 5.0 μg/mL of Hoechst33342 dye (Sigma-Aldrich) at 37°C for 90 min either alone or in combination with 15 μM Reserpine (Sigma-Aldrich), an ABC efflux pump inhibitor. 7AAD was added to the cells at a final concentration of 2 μg/mL prior to flow cytometry analysis to exclude the dead cells. The Hoechst dye was excited with UV laser and fluorescence was measured with both 675LP (Hoechst Red) and 440/40 filters (Hoechst Blue).

### Statistical analysis

Group comparisons were performed using unpaired t-tests. Prognostic analyses were performed using the log-rank test and multivariate analyses. Clinicopathological analyses were performed using Fisher's exact tests. All statistical analyses were done using GraphPad Prism 6.0 and R software. Probability values below 0.05 were considered significant.

## SUPPLEMENTARY FIGURES AND TABLES








