# Supplementary material for: Suppression of ABHD2, identified through a functional genomics screen, causes anoikis resistance, chemoresistance and poor prognosis in ovarian cancer
Source: Oncotarget. 2016 Jun 13;7(30):47620–36. doi: 10.18632/oncotarget.9951 (PMC5216966; doi:10.18632/oncotarget.9951)
Supplement: Supplementary file 1 [file oncotarget-07-47620-s001.pdf]

Suppression of *ABHD2*, identified through a functional genomics screen, causes anoikis resistance, chemoresistance and poor prognosis in ovarian cancer

SUPPLEMENTARY FIGURES AND TABLES

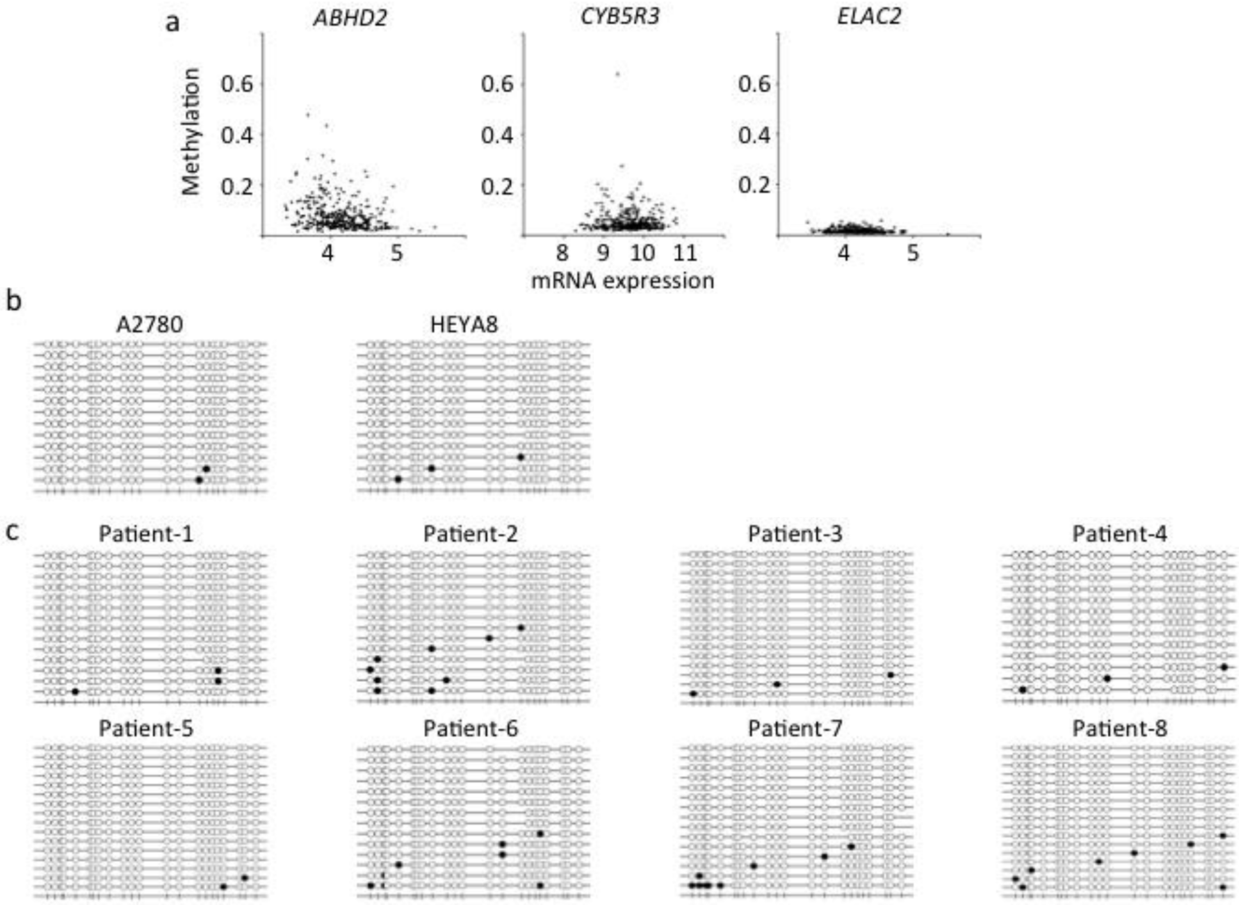

Supplementary Figure S1: Investigation of methylation status of three genes in HGSOC. a. Methylation status and mRNA expression of *ABHD2* (right), *CYB5R3* (middle) and *ELAC2* (right) in TCGA samples. Methylation was evaluated using data from the Illumina Infinium HumanMethylation27 beadchip. b,c) Methylation status detected by bisulfite sequencing of cloned alleles in the promoter region near the transcription start site of *ABHD2*. Unfilled circles indicate unmethylated CG dinucleotides, filled circles indicate methylated CG dinucleotides. **b.** Analysis of two serous ovarian cancer cell lines, A2780 (left) and HEYA8 (right). **c.** Analysis of eight HGSOC tissues.

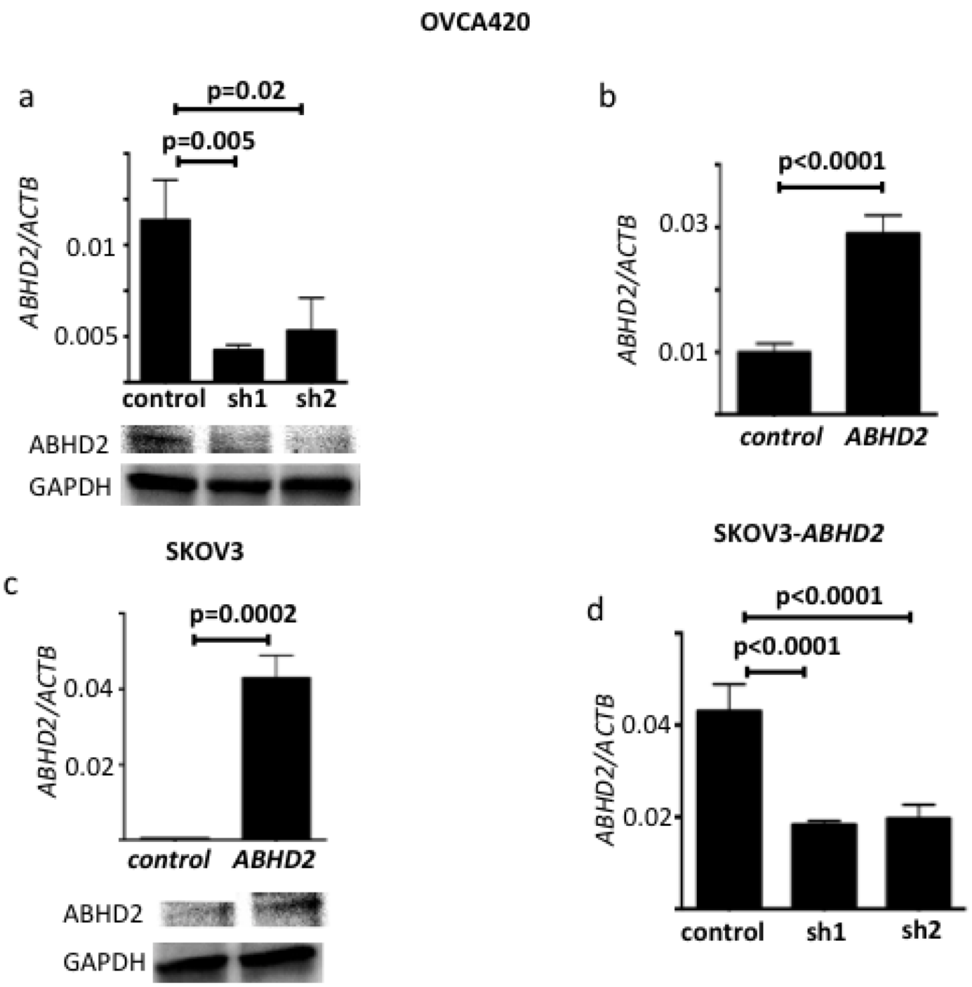

Supplementary Figure S2: Establishment of cells with suppressed or enhanced *ABHD2* expression. a. Establishment of OVCA420 cells with *ABHD2* suppression. Control; non-silencing control, sh1 and sh2; *ABHD2*-specific shRNAs. Suppression of *ABHD2* was confirmed by RT-PCR (upper) and Western-blotting (lower). **b.** Establishment of OVCA420 cells overexpressing *ABHD2* was done using transfection with a plasmid containing the *ABHD2* ORF or using a vector control plasmid. Overexpression of *ABHD2* was confirmed by RT-PCR (n=3). **c.** Establishment of SKOV3 cells overexpressing *ABHD2* was done in the same manner. Overexpression of *ABHD2* was confirmed by RT-PCR (upper) and western-blotting (lower). **d.** Additional transfection of *ABHD2*-specific shRNAs into SKOV3 cells transfected with a plasmid containing the *ABHD2* ORF. Control; non-silencing control, sh1 and sh2; ABHD2-specific shRNAs. Suppression of ABHD2 was confirmed by RT-PCR.

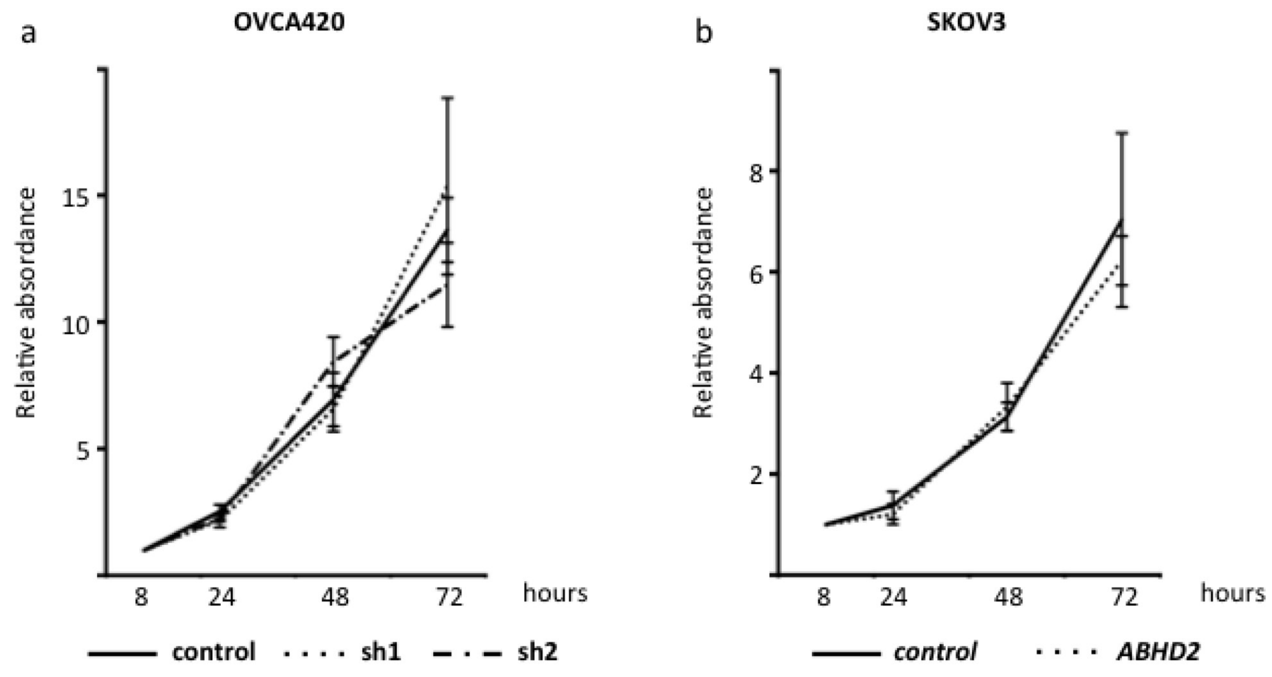

Supplementary Figure S3: Proliferation assay. a. Comparison of proliferation among the control, sh1 and sh2-OVCA420 cells *in vitro* (n=6). **b.** Comparison of proliferation between control and SKOV3-*ABHD2 in vitro* (n=6). There were no significant differences in proliferation based on ABHD2 expression status.

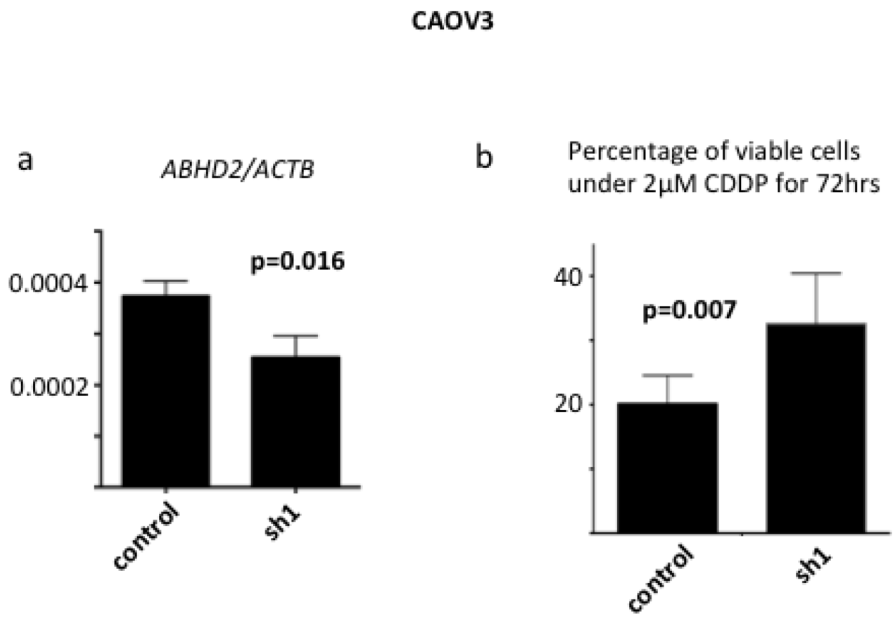

Supplementary Figure S4: Suppression of *ABHD2* causes resistance to cisplatin. a. Establishment of *ABHD2*-suppressed CAOV3 cells. Control, non-silencing control; sh1, *ABHD2*-specific shRNA. Suppression of *ABHD2* was confirmed by RT-PCR (n=3). **b.** Comparison of the percentage of viable cells between control and sh1 CAOV3 cells after treatment with 2 μM cisplatin for 72 hours (n=6). Percentage of viable cells was evaluated by WST-1 assay kit.

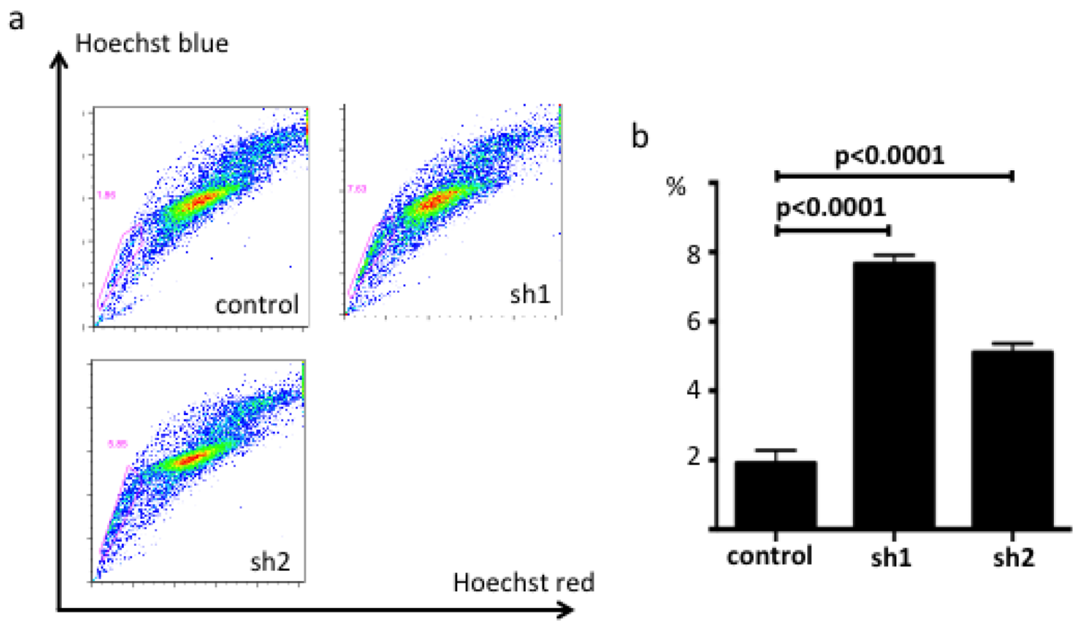

Supplementary Figure S5: Suppression of ABHD2 (sh1 and sh2) significantly increased the side population (SP) cell fraction for OVCA420 cells. a. Representative data showing the percentage of the SP fraction of control, sh1 and sh2 OVCA420 cells. **b.** Graphical representation of the data shown in panel a (n=3).

Supplementary Table S1: List of anti-sense sequences and target genes detected by 1st screening.

See Supplementary File 1

Supplementary Table S2: List of anti-sense sequences and target genes detected by 2nd screening

Anti-sense sequence	Gene symbol	NM_number
TGGTTTAAAGGGTGGGTTTGC	*A2BP1*	NM_001142333.1
CTTACGCTCCCATTGGCTCTG	*ABHD2*	NM_007011.7
AAACACTTGTTCATTTCCTGC	*BRAP*	NM_006768.3
AATCGCTGATGGGAACAGGAC	*CCL22*	NM_002990.3
AAGTAAACCTTGATGACCAGG	*CYB5R3*	NM_007326.2
AACATTAGACCAGTGCATTCG	*ELAC2*	NM_001165962.1
AATGGGAAGGAAGTGGCATGG	*PROKR1*	NM_138964.2
TTGTTAAGACTTTGTCCGTGG	*SLC30A10*	NM_018713.2
TACAGCTCAGGTTACAGAAGC	*WISP1*	NM_003882.2
TTATCCAGCATTTGTAACTGC	*KLHDC5*	NM_020782.1
AAGAAGCCATCTGTGTAGGAC	*OR2V2*	NM_206880.1

Supplementary Table S3: Primers used in this study (listed 5′ to 3′).

See Supplementary File 2

Supplementary Table S4: Characteristics of patients whose tumors were analyzed by immunohistochemistry.

See Supplementary File 3
