# Supplementary material for: Suppression of ABHD2, identified through a functional genomics screen, causes anoikis resistance, chemoresistance and poor prognosis in ovarian cancer
Source: Oncotarget. 2016 Jun 13;7(30):47620–36. doi: 10.18632/oncotarget.9951 (PMC5216966; doi:10.18632/oncotarget.9951)
Supplement: Supplementary file 2 [file oncotarget-07-47620-s002.docx]

Supplementary Table 1

List of anti-sense sequences and target genes detected by 1^st^ screening.

| Anti-sense sequence | Gene symbol | NM_number |
| --- | --- | --- |
| TGGTTTAAAGGGTGGGTTTGC | *A2BP1* | NM_001142333.1 |
| CTTACGCTCCCATTGGCTCTG | *ABHD2* | NM_007011.7 |
| TTACGCTCCCATTGGCAAATG | *ABHD2* | NM_007011.7 |
| ATTGCTTCGAATTGCATCCCG | *BBS2* | NM_031885.2 |
| AAACACTTGTTCATTTCCTGC | *BRAP* | NM_006768.3 |
| CTATCCGGTTTAGATTCAACT | *CAST* | NM_173062.1 |
| TATCCGGTTTAGATTCAACTG | *CAST* | NM_173062.1 |
| TGGTAGTTTCTCTTGTTTAGC | *COPS3* | NM_003653.2 |
| AGAGTAAAGATGGCATGGTGC | *DENND2C* | NM_198459.3 |
| TGGCAAGAGACCCAGCTGTGA | *DHRSX* | NM_145177.1 |
| TGGTTAGAGACCCAGCTGTGA | *DHRSX* | NM_145177.1 |
| TTCTATTCAGAAGGTGTGTGC | *DNAJC19* | NM_145261.2 |
| AAATCAGGCTAACTTCTTCCG | *DNAJC24* | NM_181706.4 |
| CAATTGTTATCGGTAGCTGGG | *ECD* | NM_001135752.1 |
| AATATGTTGGATGGCTTGAGG | *EIF2AK3* | NM_004836 |
| GAGTTTGATATCCCAAGGCTG | *GABBR2* | NM_005458.7 |
| TTGTTGGAGTAGGTATGATCG | *GEMIN5* | NM_015465.3 |
| TTGTGATAGTTCCATGATCGG | *GJA10* | NM_032602.1 |
| ACCACCGCATGAAGTTTGACC | *HCK* | NM_002110.2 |
| TTGGGAGACAGTAAGTGGAAG | *HEY1* | NM_001040708.1 |
| TTGTAGTTCAAAGATACTGGG | *IL18RAP* | NM_003853.2 |
| TTTCAAGCGGAATAAGACAGC | *IL19* | NM_013371.2 |
| CTCAGAGAAGGTCTCCACTGC | *KCNK7* | NM_005714.1 |
| AACCTTCTCAAAGTCTGTAGC | *KHK* | NM_006488.1 |
| GTGTGCTCTGAATAACCTGGG | *KIAA1199* | NM_018689.1 |
| TTATCATCGTCCTTGTTATCA | *KIAA1409* | NM_020818.3 |
| AAGATAGACAATCATGTCTAG | *MCHR2* | NM_001040179.1 |
| TTCTTGGTTATGTTTGGTCCG | *MDC1* | NM_014641.1 |
| ATATTAGGCTTGGAAAGCCGC | *MFN1* | NM_033540.2 |
| AAGAACTCCCAAGAATGACAC | *MGAT4C* | NM_013244 |
| TATCTCAGAGAGTGCGTCCGG | *MR1* | NM_001531.1 |
| AACAGGTACATGGGAACGTGG | *OR10A3* | NM_001003745.1 |
| AAGAGGTACATGGGCATGTGG | *OR10H5* | NM_001004466.1 |
| AAAGGAGATGTCAGTGAGAGC | *OR1J2* | NM_054107.1 |
| ATGAGAGCTAGATTTCGGAGC | *OR4D1* | NM_012374.1 |
| AGAGTCAAATGTATCTGCCAG | *OST-a* | NM_152672.5 |
| TAAGTGTCGATGAGGAAGTCG | *P2RX7* | NM_002562.4 |
| AATTTGCCGTAGGTAGTATCG | *PLK1* | NM_005030.3 |
| ATAAGGGACGATTTCCATAAG | *PPAT* | NM_002703.3 |
| AATGGGAAGGAAGTGGCATGG | *PROKR1* | NM_138964.2 |
| TTCACAGGCCACAATGATGTG | *RNASE1* | NM_198232.1 |
| TTTAATTGCCACTGTCAACTG | *SFRS5* | NM_001039465.1 |
| AAGAAGGTAATGAGGAAGCCG | *SLC2A14* | NM_153449.2 |
| TTGTTAAGACTTTGTCCGTGG | *SLC30A10* | NM_018713.2 |
| TAATTGTCGATGATGAACTGG | *SLC9A1* | NM_003047.2 |
| TACCATGAAGGCGTTCATGGG | *SOX3* | NM_005634.2 |
| TACCATGACTGCGTTCATGGG | *SOX8* | NM_014587.3 |
| TTACATTTATTTCGGGTGAGC | *SSR1* | NM_003144.3 |
| TAATTGTCGATGATGAAGTGG | *SVIL* | NM_021738.2 |
| TTACATTTATTTCCGGTGAGC | *TBCE* | NM_001079515.1 |
| TACATCTAGCATTTGCAGGGG | *TLR9* | NM_017442.2 |
| TAAGGTATTCATGTCATGACG | *TMCC3* | NM_020698.2 |
| TTTCCCAGGATGTATGTCAGG | *TRIM46* | NM_025058.3 |
| TACAGCTCAGGTTACAGAAGC | *WISP1* | NM_003882.2 |
| TATAATAGCCATTTGGACTGG | *XRCC2* | NM_005431.1 |
| ATTATGTTCCAAACCCTTATC | *ZNF568* | NM_198539.2 |
| TCTACCCTTTTTTTTCCTTCT | *ZNF578* | NM_001099694.1 |
| TTCCTGGGTGAAGTCAATAGC | *ZNF705A* | NM_001004328.2 |
| TACAATCATGTGGGTCTTGAG | *ZNF710* | NM_198526.2 |
| ATCTTCGTGTAAGTTCTGAGC | *C2CD2* | NM_015500.1 |
| ATCGTCACTATCAGGCTCAGC | *PARD6A* | NM_001037281.1 |
| AAGTAAACCTTGATGACCAGG | *CYB5R3* | NM_007326.2 |
| ATTCACAGCATTTCCCAGCAG | *PATZ1* | NM_053064.3 |
| TTGTACTTGTTCATGCTTAGG | *ST6GAL1* | NM_003032.2 |
| AACATTAGACCAGTGCATTCG | *ELAC2* | NM_001165962.1 |
| ACTCTGTTAATTTCCTGCAGC | *ANXA2P1* | NR_001562.2 |
| AATCGCTGATGGGAACAGGAC | *CCL22* | NM_002990.3 |
| TTATCCAGCATTTGTAACTGC | *KLHDC5* | NM_020782.1 |
| AAGAAGCCATCTGTGTAGGAC | *OR2V2* | NM_206880.1 |
