# Supplementary material for: Suppression of ABHD2, identified through a functional genomics screen, causes anoikis resistance, chemoresistance and poor prognosis in ovarian cancer
Source: Oncotarget. 2016 Jun 13;7(30):47620–36. doi: 10.18632/oncotarget.9951 (PMC5216966; doi:10.18632/oncotarget.9951)
Supplement: Supplementary file 3 [file oncotarget-07-47620-s003.docx]

Supplementary Table 3.

Primers used in this study (listed 5’ to 3’).

| Forward primer for subcloning | | ATCTCGACGGTATCGATGGCCGCCCCCTTCA |  |
| --- | --- | --- | --- |
| Reverse primer for subcloning | | CATGGTGGCGTCTAGACCAAGTGACGATCACAGCGAT | |
| Primer for sequencing | | GACTATCATATGCTTACCGT | |
|  | |  | |
| Primers for RT-PCR | |  | |
| Gene | |  | |
| *ABHD2* | Forward | GCCCAACATTGAATTGACCT | |
|  | Reverse | AGTTCACCATGGCTCCAAAT | |
| *CYB5R3* | Forward | ACCAGACCGAGAAGGACATC | |
|  | Reverse | TGAAGCGTGCAGAATGTTTG | |
| *ELAC2* | Forward | TCCATTGAAAGGAATAGAACTGG | |
|  | Reverse | CTGTCATGGTTTCATCCTCGT | |
| *ACTB* | Forward | CCAACCGCGAGAAGATGA | |
|  | Reverse | CCAGAGGCGTACAGGGATAG | |
|  | |  | |
| Primers for Bisulfite-PCR | |  | |
| Forward | | GGATAGTGGGATAATAATTGAGGTT | |
| Reverse | | ACTTTAATCCAAAAACCCAAA | |
