# Supplementary material for: Suppression of ABHD2, identified through a functional genomics screen, causes anoikis resistance, chemoresistance and poor prognosis in ovarian cancer
Source: Oncotarget. 2016 Jun 13;7(30):47620–36. doi: 10.18632/oncotarget.9951 (PMC5216966; doi:10.18632/oncotarget.9951)
Supplement: Supplementary file 4 [file oncotarget-07-47620-s004.docx]

Supplementary Table 4

Characteristics of patients whose tumors were analyzed by immunohistochemistry.

1. HGSOC patients.

| Age (y) | median | 56.4 |
| --- | --- | --- |
|  | range | 28-81 |
|  |  | Number |
| FIGO stage | Ia | 4 |
|  | Ic | 1 |
|  | II a | 2 |
|  | II c | 2 |
|  | IIIb | 5 |
|  | IIIc | 19 |
|  | IV | 3 |
|  | Total | 36 |

1. SBT patients

| Age (y) | median | 50.9 |
| --- | --- | --- |
|  | range | 28-82 |
|  |  | Number |
| FIGO stage | Ia | 4 |
|  | Ib | 1 |
|  | Ic | 1 |
|  | II b | 1 |
|  | IIIb | 1 |
|  | Total | 8 |

1. Normal fallopian tube patients.

| Age (y) | median | 48.6 |
| --- | --- | --- |
|  | range | 40-62 |
| Diagnosis | | Number |
| Leiomyoma | | 4 |
| Corpus cancer | | 3 |
| Ovarian cyst | | 2 |
| CIN | | 2 |
| Cervical cancer | | 1 |
| Total | | 11 |
